# Acquired spherocytosis in the setting of myelodysplasia

**DOI:** 10.1016/j.lrr.2022.100332

**Published:** 2022-06-02

**Authors:** Linda Katharina Karlsson, Mathis Nygaard Mottelson, Jens Helby, Jesper Petersen, Andreas Glenthøj

**Affiliations:** Department of Hematology, Copenhagen University Hospital - Rigshospitalet, Copenhagen, Denmark

**Keywords:** Spherocytosis, MDS, Hemolysis, Acquired hemolysis, Splenectomy

## Abstract

Hereditary spherocytosis (HS) is the most prevalent red blood cell (RBC) membrane disorder. We report a rare case of acquired *SPTB* spherocytosis coinciding with a myelodysplastic syndrome associated *U2AF1* mutation, neither found in germline DNA. The diagnosis was confirmed by Eosin-5-Maleimide binding assay and Next Generation Sequencing (NGS). The patient recovered quickly after splenectomy, which confirms that his myelodysplastic syndrome (MDS)-associated *U2AF1* mutation did not affect the clinical picture. This case highlights the essence of thoroughly examining the etiology of hemolytic indices, despite bone marrow morphology and myeloid gene panel supporting a diagnosis of MDS with single line dysplasia.

## 1 Key points

In elderly, acquired erythrocyte membrane disorders may explain anemia in patients suspected of myelodysplasia.

## 2 Introduction

Myelodysplastic syndrome (MDS) is an acquired clonal stem cell disorder, mostly seen amongst the elderly population [1]. It is characterized by ineffective hematopoiesis and progressing cytopenias [2]. The clinical presentation is heterogeneous, and most commonly includes symptoms of anemia [1]. Other symptoms include bleeding and infections, as well as a propensity to transform into acute myeloid leukemia [1,2]. The diagnosis relies on bone marrow dysplasia, complemented by cytogenetic abnormalities [3]. Cytogenetics is increasingly complemented by genetic screening tools such as Next Generation Sequencing (NGS) or Whole Genome Sequencing, enabling identification of a large number of genes associated with MDS [4,5]. The impact of most single somatic mutations is yet unclear and many are age-associated [1,3].

Hereditary spherocytosis (HS) is the most prevalent red blood cell (RBC) membrane disorder in Caucasians [6]. Inherited genetic mutations or deletions in the genes of erythrocyte membrane components cause hemolytic anemia due to weakened vertical linkage between the RBC membrane and the underlying cytoskeleton, leading to reduced erythrocyte surface area, causing RBC retention and destruction in the spleen [6]. Clinically, patients with HS display variable degrees of anemia, reticulocytosis, jaundice, and splenomegaly [6,7]. Gallstones are common due to increased bilirubin excretion, and hemolytic crises and aplastic anemia are associated with viral infections [6,7]. The diagnosis often relies on functional testing of the RBC membrane by Eosin 5-Maleimide (EMA) binding assay or osmotic gradient ektacytometry [8].

In this report, we present a rare case of acquired spherocytosis coinciding with an MDS-associated mutation.

## 3 Case description

A 71-year-old man was referred to a gastroenterological clinic due to macrocytic anemia (hemoglobin 9.9 g/dl, mean corpuscular volume 107 fL) and hyperbilirubinemia (4.4 mg/dL). Haptoglobin was absent (<0.08 g/L) and lactate dehydrogenase was normal (187 U/L). Serum folate levels had previously been low, and the patient was taking iron supplements and folic acid. An ultrasound of the abdomen showed gallstones and splenomegaly. Morphological evaluation of bone marrow biopsy and aspirate showed single-lineage dysplasia (SLD) of >10% of the erythroid precursors, which was found compatible with MDS-SLD if alternative diagnoses such as hemolysis could be excluded. Cytogenetics was not performed.

**Fig. 1:**
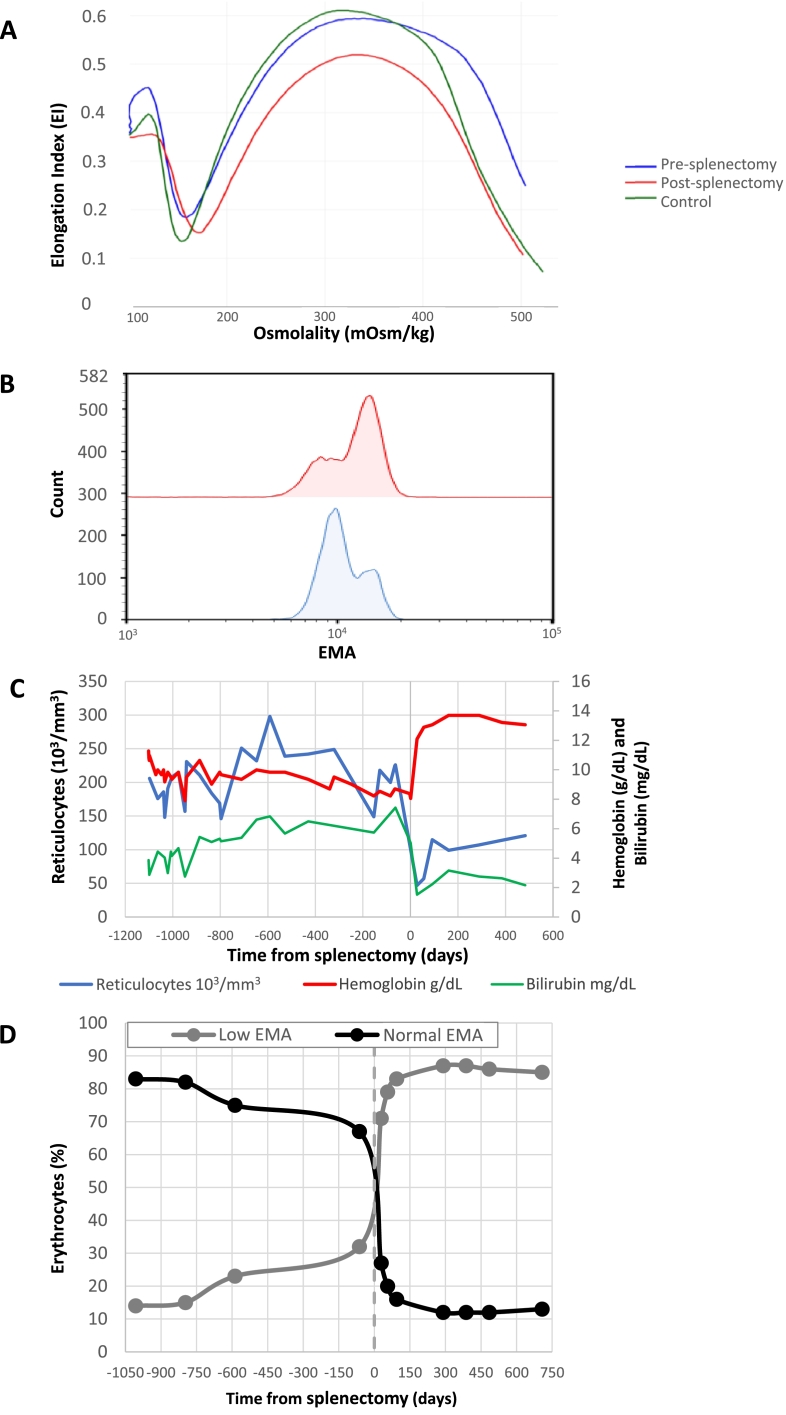
(A) Ektacytometry before and after splenectomy. (B) Eosin 5-Maleimide (EMA) binding assay two months prior to splenectomy (top curve) and two months post splenectomy (bottom curve). (C) Hemoglobin (g/dL), bilirubin (mg/dL), and reticulocytes (10^3^/mm3) before and after laparoscopic splenectomy (day 0). Blood transfusions with one or two units of blood were given on day −955, −948, and −832. (D) Development in the proportion of erythrocytes expressing low or normal levels of EMA binding.

**Fig. 2:**
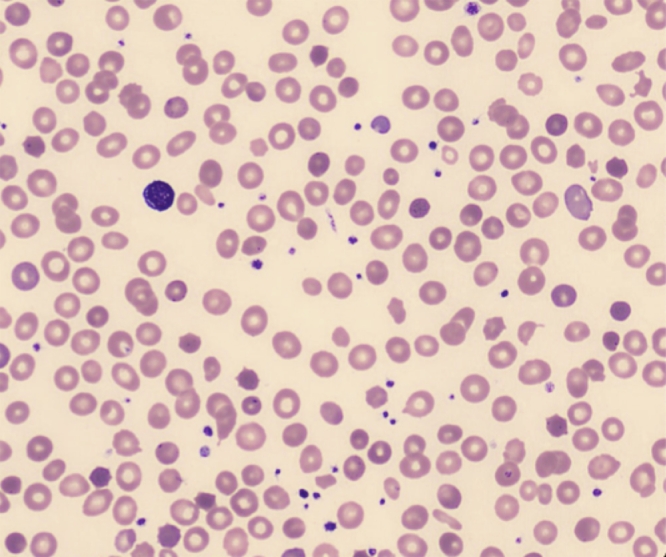
Peripheral blood smear with spherocytes acquired with a CellaVision DM1200 analyzer.

The patient was referred to a hematological clinic where a CT scan of the chest, neck and abdomen showed a slightly enlarged spleen (13.9 × 6.5 cm). Direct anti globulin test and a flow cytometric direct anti globulin test were both negative and cold agglutinins were not present. Genetic testing for Gilbert's syndrome and pyruvate kinase deficiency as well as flowcytometric testing for paroxysmal nocturnal hemoglobinuria were also negative. Osmotic gradient ektacytometry was initially negative for hereditary spherocytosis (Fig. 1A) and the small pathological erythrocyte population on the EMA-binding test was initially discarded by the laboratory as an artefact (Fig. 1B). A myeloid neoplasm NGS performed on DNA from peripheral blood cells revealed an MDS associated *U2AF1* mutation (NM_006758.2: c.470A>*G*) with an allele burden of 24%. Careful examination of the peripheral blood smear (Fig. 2) revealed numerous spherocytes. This prompted a hemolysis NGS panel which demonstrated a *SPTB* mutation (NM_001024858.2: c.398T>*G*), associated with hereditary spherocytosis. The allele burden was 31% at the time of diagnosis and increased to 42% three years later. The *SPTB* mutation was not found in germline DNA obtained from cultivated fibroblasts. Repeated EMA-binding tests exposed two different erythrocyte populations, whereof one indicated spherocytosis (Fig. 1B). The EMA binding was not reduced for the normal fraction of erythrocytes whereas the pathological population consistently had a decrease of >15% compared to normal controls. The patient was thus diagnosed with acquired spherocytosis, whereas the MDS associated *U2AF1* could likely be attributed to clonal hematopoiesis without effect on the patient's hemolytic anemia.

The patient received three units of blood in the setting of surgery and one unit due to chronic fatigue. The patient underwent splenectomy due to persistent anemia with hemoglobin levels around 8.0–9.7 g/dL, need of blood transfusions, and a declining performance status. This resulted in reduced hemolysis with normalization of hemoglobin levels around 13.1 g/dL and bilirubin levels around 2.3 mg/dL (Fig. 1C). Follow-up 2 months after splenectomy showed persistent, close to normalized hemoglobin, and the patient reported substantially improved quality of life.

## 4 Methods

The patient consented to the publication of the included data. Patient data was collected retrospectively via the review of electronical hospital medical charts.

### 4.1 EMA-binding test

The EMA-binding test was performed on EDTA-stabilized blood within 48 h of sampling [8]. The labeling of RBCs with EMA, usage of mid-range FL1 Rainbow Fluorescent Particles (BD Biosciences, NJ, USA) and flow cytometry was performed [8,9].

### 4.2 Targeted next-generation sequencing (tNGS)

DNA was extracted using QIAamp DNA Blood Mini QIAcube Kit (Qiagen, Hilden, Germany). Two different NGS panels were employed:

Hemolysis: *SPTA1, SPTB ANK1 SCL4A1 EPB41 EPB42, PIEZO1, G6PD, PKLR, HK1, GPI, NT5C3, PKGK1, TPI1, GSS, GSR, ADA, AK,1PFKM, PFKL, GATA1, KLF1*. Targeting, amplification and normalization was performed using the manufacturer′s instructions (TruSeq Custom Amplicon v1.5, Illumina, CA, USA). Sequencing was done on a MiniSeq (Illumina) via MiniSeq Mid Output Kit (300x paired-end; Illumina). The BaseSpace Variant Interpreter (Illumina) and Integrative Genomics Viewer Software [10] were used for Sequencing analyses. Variants were called with at least 10 variant reads, a minimum read depth of 30x, and classified in categories according to recommendations from the American College of Medical Genetics and Genomics [11], using Base Space Variant Interpreter (Illumina). Allele burdens were determined based on a read depth of at least 600x.

Myeloid: *RUNX1, KMT2A, TET2, ASXL1, IDH1, IDH2, CSF3R, SRSF2, SETBP1, ETNK1, SF3B1, EZH2, U2AF1, JAK2, MPL, CALR, ZRSR2, TP53, DNMT3A.* Detection of genetic variants of relevance for MDS using NGS was performed using the Ion Torrent S5XL sequencer (Thermo Fisher Scientific) following the manufacturer's protocol. The sequenced library was prepared using primer pools designed to amplify all coding exons of all the included genes. Data was aligned against hg-19, and analyzed via IR v.5.4, MDS test workflow version 5.4, with LOD of 5%, minimum allele coverage of 100x and filtered through the UCSC common SNP filter.

## 5 Results and discussion

In this article, we report a rare case of acquired spherocytosis in a patient suspected of MDS, without familiar history of hemolytic anemia. The patient had previously been well and reported that hemoglobin measurements from the 1990s sampled before simple surgery had been normal. However, this could not be confirmed as these blood tests were no longer available due to the implementation of new electronical medical charts (replacing paper-based charts), which do not contain older data than what is presented in Fig. 1C. The absence of germline DNA mutations and swift recovery after splenectomy confirm that the condition was acquired and that the MDS associated *U2AF1* mutation did not affect the clinical picture including hemoglobin levels. In accordance with increased spherocyte longevity post-splenectomy, repeated EMA-binding test demonstrated a rapid and sustained increase in the spherocyte fraction (Fig. 1D).

The patient had gallstones and splenomegaly at the time of diagnosis, both associated with hemolysis.

The diagnosis was delayed as only one of the two erythrocyte populations were recognized on the first EMA-binding test due to overlap (Fig. 1B). Careful examination of the peripheral blood smear is still a valued tool and was in this case instrumental in guiding the diagnostic workup towards HS, which was finally confirmed by NGS. This case highlights the essence of thoroughly examining the etiology of hemolytic indices, despite bone marrow morphology and myeloid gene panel supporting a diagnosis of MDS with single line dysplasia.

## Informed consent

The patient consented to the publication of the patient details published in this Case Report.

## Declaration of Competing Interest

None of the authors reported any conflicts of interest.
